# Vascular access type and mortality in haemodialysis: a retrospective cohort study

**DOI:** 10.1186/s12882-020-01889-4

**Published:** 2020-06-18

**Authors:** Dieter De Clerck, Florence Bonkain, Wilfried Cools, Patricia Van der Niepen

**Affiliations:** 1 Department of Nephrology & Hypertension, Vrije Universiteit Brussel (VUB), Universitair Ziekenhuis Brussel (UZ Brussel), Laarbeeklaan 101, 1090 Brussels, Belgium; 2 Vrije Universiteit Brussel (VUB), Interfaculty Center Data processing and Statistics, Laarbeeklaan 103, 1090 Brussels, Belgium

**Keywords:** Haemodialysis, Vascular access, AV fistula, Catheter, Survival, Mortality

## Abstract

**Background:**

Haemodialysis patients have a high mortality rate. Part of this can be attributed to vascular access complications. Large retrospective studies have shown a higher mortality in patients dialysed with a catheter, which is mostly ascribed to infectious complications. Since we observe very little infectious complications in our haemodialysis patients, the aim of our study was to assess if we could still detect a difference in survival according to vascular access type.

**Methods:**

Patients that started chronic haemodialysis treatment between 1/1/2007 and 31/12/2016 at the ‘Universitair Ziekenhuis Brussel’ were retrospectively studied. The time to death was studied as a function of the two main vascular access types using survival analysis, considering the type of vascular access at the initiation of dialysis or as time varying, and accounting for the available baseline characteristics.

**Results:**

Of 374 patients 309 (82.6%) initiated haemodialysis with a catheter, while 65 patients initiated with an arteriovenous access. Vascular access type during follow-up did not change in 74% of all patients. A Kaplan Meier plot did not suggest a survival dependent on the vascular access type at start. An extended cox proportional hazard analysis showed that vascular access type was not independently correlated with mortality. However, age, history of congestive heart failure and active cancer at initiation of dialysis were independently associated with mortality.

**Conclusions:**

In this retrospective cohort study, haemodialysis vascular access type was not independently correlated with patient survival, even after taking into account change of vascular access over time.

## Background

Chronic haemodialysis patients have a high mortality, mostly due to cardiovascular and infectious diseases [1, 2]. Part of this high mortality rate is ascribed to haemodialysis vascular access–related complications [3]. Permanent vascular access consists either of arteriovenous (AV) fistulas or grafts, either of tunneled cuffed dialysis catheters. It is known that the use of dialysis catheters is associated with a higher risk for bacteremia [4]. Their use is also associated to an inflammatory state and hence increased cardiovascular risk [5]. Arteriovenous fistulas or grafts on the other hand are associated with cardiac remodeling and can induce or aggravate heart failure [6]. In general it is accepted that the infectious risk of dialysis catheters outweighs the cardiac risk of AV fistulas or grafts. Hence the policy on vascular access in haemodialysis patients is to promote the use of an AV fistula or graft, unless there is severe heart failure, access induced limb ischemia or a limited prognosis [7]. The European dialysis working group (EUDIAL) recently proposed a patient-centered approach instead of a fistula first policy in the elderly, though still suggesting that an AV fistula should be the first choice for the majority of elderly patients [8]. In our centre and by extent in Belgium and Europe there is a high use of dialysis catheters, with even a decreasing trend to use an AV fistula, and an increasing trend to use a catheter [9]. The complication rate from this high catheter use seems to vary importantly though. Large differences in catheter related bloodstream infections (CRBI) are described in the literature. To our knowledge the lowest reported CRBI rate can be found in a recent publication by El-Hennawy and colleagues with a CRBI rate of 0.17 per 1000 catheter days [10]. Since we witness in our centre an even lower CRBI rate (as low as 0.14 CRBI per 1000 catheter days), the objective of our study was to compare patient outcome according to the vascular access type in a dialysis population with very little infectious complications from catheters.

## Methods

### Study population

We retrospectively studied all patients who initiated chronic haemodialysis treatment at the university hospital ‘Universitair Ziekenhuis Brussel’ in Brussels, Belgium, between 1/1/2007 and 31/12/2016. Chronic haemodialysis treatment was defined as every haemodialysis treatment initiated in patients with end stage renal disease (ESRD) with the intention of being a chronic treatment, as well as every haemodialysis treatment for acute renal failure that was continued for more than 6 weeks. We only included patients who have not been treated by renal replacement therapy (haemodialysis, peritoneal dialysis or transplantation) before.

Part of the data was extracted from the Flemish registry NBVN (Nederlandstalige Belgische Vereniging voor Nefrologie): date of birth, gender, date of first dialysis, type of kidney disease, comorbidities at initiation of dialysis (diabetes mellitus, congestive heart failure, ischemic heart disease, cerebrovascular disease, peripheral vascular disease, and malignancy) and in case of death, cause of death. From the medical records, the following data were added: the vascular access type at start and every change in vascular access type during the study period, the presence of a regular follow-up at the outpatient clinic (defined as first visit more than 3 months before dialysis initiation and last visit not longer than 12 months before dialysis initiation), if the patient initiated dialysis at the intensive care unit and whether dialysis was initiated because of ESRD related complications or other pathology, the presence of cancer (multiple myeloma or other) at the initiation of dialysis, blood values at initiation of dialysis (albumin, hemoglobin, C-reactive protein [CRP], creatinine, estimated glomerular filtration rate [eGFR]) and finally the outcome at the end of the observation (still on haemodialysis, deceased, transplanted, transferred to another centre, transferred to peritoneal dialysis, lost to follow-up, recovery of kidney function, decision to stop dialysis by the patient or nephrologist).

### Statistics

The analysis starts with a general description of the data, including Kaplan Meier plot. The time to death, starting at the beginning of a treatment, is modeled using an (extended) cox proportional hazard model. The main variable of interest is the vascular access type, either catheter or arteriovenous access. In addition to the vascular access type, various predictors were included in the model, such as several blood values and comorbidities. Because of the intra patient changes in vascular access type this variable was also considered as time varying.

The model at a minimum considered the vascular access type and time to death. Other predictors were evaluated and retained if significant using a manual forward and backward selection based on a Wald test. In each step, the inter relations between variables were monitored to avoid selecting models that showed multicollinearity.

## Results

In total, 374 patients were included in the study. Patient characteristics are shown in Table 1. Mean age at initiation of dialysis was 69 years (23.5% of patients were 80 years or older). Two thirds (66%) of patients were men. Of the 374 patients 83% initiated dialysis with a catheter: 62% were temporary catheters, 38% were tunnelled cuffed catheters (TCC). All catheters were double lumen catheters. Only 17% initiated with an AV access (the vast majority with an AV fistula - only 1 patient initiated with an AV graft): 31% were wrist fistulas (all radial artery-to-cephalic vein), 69% were upper arm fistulas (46% brachial artery-to-cephalic vein, 18% brachial artery-to-basilic vein, 5% brachial artery-to-median cubital vein). The mean time between the construction of the AV access and the initiation of dialysis was 259 days (median time 171 days, range 17 tot 2437 days).

**Table 1:** Description of the study population, according to vascular access type at dialysis initiation

	total	catheter	AV access
Total number of patients	374	309	65
Male (%)	66.3	64.7	73.8
Mean age at dialysis initiation (years)	69 (21–95)	68,3 (21–95)	69,9 (37–85)
Patients without regular follow-up (%)	38.6	45.9	4.6
Causes of ESRD (%)			
Diabetes	28.3	28.5	27.7
Hypertension/vascular	27.8	26.9	32.3
Glomerulonephritis	7.2	7.4	6.2
ADPKD	6.1	4.2	1.5
Malignancy	5.3	5.8	3.1
Obstructive/urologic	3.7	3.9	3.1
Other known cause	12	13.9	3.1
Cause unknown	9.4	9.4	9.2
Comorbidities (%)			
Diabetes	48.1	48.2	47.2
Ischemic heart disease	30.4	30.7	28.8
Peripheral vascular disease	26.2	27.6	19.2
Congestive heart disease	24.9	26.5	17.3
Cerebrovascular disease	15.9	15.2	19.2
Cancer at start of dialysis	8.8	10.3	1.5
Multiple myeloma	3.5	4.2	0
Dialysis started at ICU	24.3	28.1	6.2
Because of a non-ESRD related illness	11.8	13.3	4.7
Mean (SD) blood values at dialysis initiation			
Albumin (g/dl)	33.8 (6.3)	33.2 (6.4)	36.8 (4.3)
Hb (g/dl)	9.9 (1.7)	9.7 (1.6)	10.6 (1.7)
CRP (mg/dl)	47.8 (72.9)	51.3 (75.4)	31.3 (57.7)
Creatinine (mg/dl)	6.6 (3.3)	6.6 (3.4)	6.6 (2.6)
eGFR (ml/min/1.73 m^2^)	9.4 (5.7)	9.6 (6.1)	8.4 (3.2)
Causes of death (%)			
Cardiovascular	31.0	32.0	27.3
Infectious	22.8	22.4	24.2
Malignancy	7.6	8.8	3.0
Decision to stop dialysis	6.3	5.6	9.0
Haemorrhage	5.1	5.6	3.0
Other known cause	12.0	11.2	15.2
Unknown	15.2	14.4	18.2

Patients initiated dialysis with a catheter instead of an AV access for several reasons: almost half of the patients initiating dialysis with a catheter were patients without a regular follow-up (46%), as compared to patients initiating with an AV access, where almost all patients had a regular follow-up at the outpatient clinic. Most of these patients initiating with a catheter had to start dialysis within 3 months after their first contact with a nephrologist. The reasons why patients initiated with a catheter despite a regular follow-up were various: a sudden deterioration of kidney function in a previously stable CKD patient (12%), a contraindication for an AV access (mostly severe heart failure) (9%), the AV access was not usable at the time of dialysis initiation (6%), the patient previously chose peritoneal dialysis as a dialysis modality, but instead initiated haemodialysis (5%), the patient previously chose conservative care, but finally chose to initiate dialysis (3%), the decision not to place an AV access because of very old age (2%). In 17% of the patients an AV access theoretically could have been placed in time, but the time of AV access creation was either postponed by the nephrologist or the patient, or delayed due to the waiting time for predialysis education, AV access work-up or surgery.

During follow-up 74% of patients retained the same vascular access type. On the other hand, vascular access type was changed to a fistula in 20% of patients who initiated with a catheter (including 6% who eventually change back to a catheter), and to a catheter in 6% of patients who initiated with a fistula (including 2% who eventually changed back to a fistula).

Besides the difference in follow-up at the outpatient clinic as mentioned above, patients initiating dialysis with a catheter more often started at the ICU as compared to patients initiating with an AV access (28 and 6% respectively). In about half of patients starting at the ICU, this was because of an ESRD related complication, as hyperkalaemia or pulmonary oedema.

Another difference was the presence of cancer, which was present at the time of dialysis initiation in 10% of patients that initiated with a catheter, while this was only the case in 1.5% of patients with an AV access.

The most frequent causes of ESRD were diabetes (28%), hypertension/vascular (28%), glomerulonephritis (7%), autosomal dominant polycystic kidney disease (ADPKD) (6%), malignancy (5%), and obstructive/urologic problems (4%).

Comorbidities were only known in 309 (83.6%) patients. Diabetes mellitus was present in 48% of patients, ischemic heart disease in 30%, peripheral vascular disease in 26%, congestive heart failure in 25%, and cerebrovascular disease in 16%. Peripheral vascular disease and congestive heart failure were more often present in the group of patients that initiated with a catheter.

At initiation of dialysis, mean eGFR (CKD-EPI formula) was 9.4 ml/min. Mean CRP was 47.8 mg/dl, mean albumin 34 g/dl and mean Hb 9.9 g/dl. Patients that started with a catheter had already a higher inflammatory state (higher CRP, lower albumin and Hb) at dialysis initiation than patients starting with an AV access.

The mean time of follow-up was 2.1 years (range 1 day to 10.8 years). During the observation period, 158 patients out of 374 died (42%). Only 69 patients (18%) were still on dialysis in our centre at the end of the study. The other patients either were transplanted (11%), transferred to another dialysis centre (10%), switched to peritoneal dialysis (6%) or recovered renal function allowing them to stop dialysis (6%). In 5% of patients dialysis was stopped either because the patient decided to stop, or the nephrologist decided to end treatment (for instance because of dementia); this decision may or may not have led to the death of the patient. Only 1% of patients were lost to follow-up.

The most frequent causes of death were cardiovascular (31%), infectious (23%), malignancy (8%), the decision to stop dialysis (6%) and haemorrhage (5%). Only one death (0.63%) was highly suspicious of being caused by a dialysis catheter infection, and this was in a patient who refused being hospitalized for treatment.

**Fig. 1:**
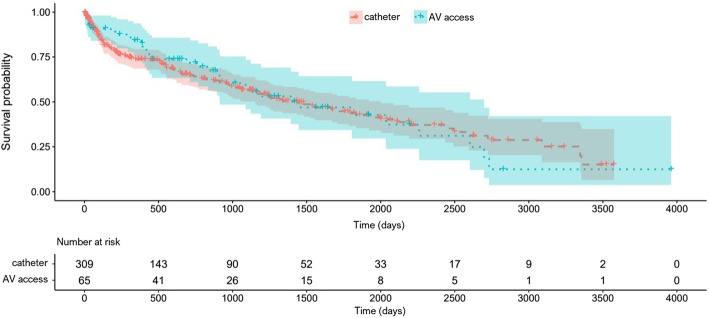
Kaplan Meier analysis comparing dialysis patients with a catheter with patients with an arteriovenous access, at dialysis initiation

Overall 5-year mortality of our study population was 56.5%. No difference in mortality between the patients that initiated dialysis with an AV access compared to patients that initiated with a catheter was observed (hazard ratio for mortality in patients with an AV access was 0.98 with 95% confidence intervals 0.67 to 1.44). In the multivariate analysis patient age, a history of congestive heart failure and active cancer at dialysis initiation were independently associated with a higher mortality. There was a trend for CRP to be associated with mortality, but this was not statistically significant. Finally, vascular access type at dialysis initiation was not associated with mortality in this multivariate analysis (Table 2). Survival of patients based on their vascular access at dialysis initiation is presented in a Kaplan Meier plot (Fig. 1).

**Table 2:** Unadjusted and adjusted rates of mortality based on vascular access type at baseline

	HR (95% CI)	p
**Univariate analysis**		
Dialysis access		
*Catheter (reference)*		
*AV access*	0.98 (0.67–1.44)	0.921
**Multivariate analysis**^**a**^		
Dialysis access		
*Catheter (reference)*		
*AV access*	1.30 (0.82–2.04)	0.264
Age	1.05 (1.03–1.07)	< 0.001
Congestive heart disease	2.02 (1.37–2.98)	< 0.001
Active cancer	2.49 (1.37–4.50)	0.003
CRP	1.003 (1.000–1.005)	0.018

^a^ Multivariate analysis adjusted for age, sex, diabetes, cancer, congestive heart disease, dialysis initiation at the ICU, CRP and albumin

When we only examined the group of patients that kept the same vascular access type during the study, we were not able to show a significant difference in mortality between patients with an AV access and patients with a catheter either.

When mortality was compared based on vascular access as a variable that changes over time, the hazard ratio for mortality was 0.61 (*p* = 0.005, 95% CI 0.44–0.87) for AV access. However, in the multivariate analysis the difference between AV access and catheter was much smaller and no longer statistically significant (hazard ratio 0.92, *p* = 0.722, 95% CI 0.58–1.46). Age, history of congestive heart failure and active cancer at dialysis initiation were still associated with mortality in this analysis (Table 3).

**Table 3:** Unadjusted and adjusted rates of mortality based on vascular access type as a variable changing over time

	HR (95% CI)	p
**Univariate analysis**		
Dialysis access		
*Catheter (reference)*		
*AV access*	0.61 (0.44–0.87)	0.005
**Multivariate analysis**^**a**^		
Dialysis access		
*Catheter (reference)*		
*AV access*	0.92 (0.58–1.46)	0.722
Age	1.04 (1.02–1.06)	< 0.001
Congestive heart disease	1.88 (1.27–2.76)	0.001
Active cancer	2.93 (1.62–5.29)	< 0.001
CRP	1.004 (1.001–1.006)	0.003

^a^ Multivariate analysis adjusted for age, sex, diabetes, cancer, congestive heart disease, cerebrovascular disease, dialysis initiation at the ICU, CRP, albumin, eGFR and Hb

We also compared patients initiating dialysis with a TCC to patients initiating with a temporary catheter. We found no significant difference in mortality between both groups. The unadjusted rate for mortality was 0.85 (95% CI 0.57–1.28) for patients initiating with a TCC compared to patients initiating with a temporary catheter.

## Discussion

Many studies on the relation between vascular access and mortality in haemodialysis patients have been published over the last 20 years. Dhingra and colleagues were the first to show in 2001 that in a prevalent USA haemodialysis population of more than 5500 patients the use of dialysis catheters was associated with a 54 to 70% higher mortality risk (depending on the presence or absence of diabetes) compared to dialysis with an AV fistula, after correction for several variables [11]. In the following years several other studies were published based on very large cohorts from the USA, Australia, New Zealand and Canada, confirming the relation between catheter use and mortality [12–17]. Only few European studies were published on this subject. Di Iorio and colleagues published in 2004 a multicentre study including 2201 prevalent and 635 incident haemodialysis patients. A higher mortality rate in catheter users compared to fistula users abolished after correcting for age, gender, malnutrition, diabetes, hemoglobin, albumin and comorbidity in both patient groups [18]. The study of Di Iorio et al. is to our knowledge the only one until now that did not show an independent relation between vascular access type and mortality. In contrast, the study by Pisoni and colleagues in 2009 on the DOPPS data from several European countries, Japan, Australia, New Zealand, Canada and the USA, showed again an independent correlation between vascular access and mortality, with a 32% higher mortality rate in patients dialyzed with a catheter compared to an AV fistula [19].

A problem though with these observational data on the subject of vascular access is the question whether there is residual bias, in particular bias due to treatment by indication. The nephrologist’s decision to initiate a patient on dialysis with a catheter or an AV access is not a random choice, but is guided by several other variables, such as age, comorbidities, prognosis, and late referral. Multiple statistical methods such as marginal structural models, instrumental variables and propensity scores have been applied in order to deal with this potential bias, but the question still remains if there are no confounders left influencing the relation between vascular access and mortality [13, 19, 20]. The idea remains that a patient that starts or continues dialysis with a catheter is intrinsically more ill than a patient that is able to get an AV access. Some more recent studies are in favour of this hypothesis. A study by Brown and colleagues on more than 115,000 patients over 67 years old from the US Renal Data System showed that patients initiating dialysis with a fistula had a 50% lower mortality risk at 58 months, compared to patients initiating with a catheter [21]. However, patients initiating with a catheter after a failed fistula attempt also had a 34% lower mortality risk. This led them to conclude that at least two thirds of the mortality benefit in patients with a fistula was due to patient factors and not due to the vascular access itself. Ravani and colleagues examined the DOPPS data from 1996 to 2011 on the relation between vascular access type, vascular access complications and mortality, and concluded that the relation between vascular access type and mortality could not be explained by vascular access type complications [22]. Finally, Ko and colleagues showed recently that in patients over 80 years old, survival in patients initiating with a catheter and switching to a fistula within the first year of dialysis is comparable to patients initiating immediately with a fistula, as opposed to patients that stay with a catheter and have a worse outcome [23]. This again suggests that other patient factors might influence the association between vascular access and outcome.

Our observational single centre study may add some interesting elements to the already existing literature on vascular access and mortality. The added value of our study consists of the detailed information on the vascular access of the patients during their entire follow-up, as opposed to most studies where only information on the vascular access type at start of the study is available, and what happens afterwards with vascular access is ignored. Therefore we were able to perform an extended Cox proportional hazard analysis with vascular access type as a variable that changes over time. Second, to our knowledge this is the first study that tried to show a difference in mortality related to vascular access in a dialysis population with a CRBI rate in the very low range of the spectrum, which we consider the most important complication of the use of dialysis catheters and hence the most important factor influencing morbidity and mortality. In our study dialysis with an arteriovenous access is associated with a 39% lower mortality risk as compared to dialysis with a catheter. However, after correction for several other variables like age, several comorbidities, dialysis initiation at the ICU and baseline blood values such as albumin, CRP, Hb and eGFR, this correlation between vascular access and mortality is no longer significant, whereas age, congestive heart failure, cancer and CRP still remain significantly correlated with mortality. Therefore this study suggests that the worse outcome associated with dialysis catheters might be due to other patient factors and not due to the dialysis access itself.

The very low incidence of CRBI in our dialysis centre might certainly play a role in this observation. The dialysis team has had a special interest in prevention of infection since a very long time. We are convinced that the low infection rate is the result of a continuous follow-up on all different aspects of catheter management, including the sterile placement of the catheter, the aseptic handling of the catheter by the dialysis nurse and patient education. We believe that in particular the manipulation of the catheter by the dialysis nurse plays an important role: therefore we continue to invest in education on hygiene and aseptic technique, as well as in easy to handle materials and sufficient work space. The type of catheter locking solution that we used over the years might play a role as well, because of its antimicrobial effect: the heparin lock was replaced by the citrate 30% locking solution in Juli 2008. This was used until August 2012, when it was replaced by the taurolidin + citrate 4% + heparin 500 IU/ml locking solution. An antimicrobial ointment was not used during the study period, but was introduced afterwards.

A limitation of our study is the limited number of patients and therefore a statistical difference between both groups might have been missed. Moreover, as this is a single-centre study, these findings cannot be extrapolated to other dialysis centres, where there might be a different vascular access complication rate, influencing the outcome of the patient.

## Conclusions

Until there are more prospective data, our aim will still be to promote as much as possible the use of an arteriovenous access in our patients, unless the patient has severe heart failure or a limited prognosis due to very old age or severe comorbidities. With the current data however we feel more comfortable to continue our current policy to promote an AV access in the majority of our patients, but without pushing patients with a limited prognosis or a relative contra-indication to get an AV access at all cause. In these patients a tunnelled catheter might be a worthy alternative.

## Data Availability

The data that support the findings of the study are not publicly available due to privacy restrictions (the data contains information that could compromise the privacy of the study participants).

## Abbreviations

ADPKD: Autosomal dominant polycystic kidney disease
AV: Arteriovenous
CRBI: Catheter related bloodstream infection
CRP: C-reactive protein
eGFR: Estimated glomerular filtration rate
ESRD: End stage renal disease
Hb: Hemoglobin
ICU: Intensive care unit
TCC: Tunnelled cuffed catheter
